# Limitations in the Assessment of Pain Reporting Reliability—Validation of the Focused Analgesia Selection Test

**DOI:** 10.1111/papr.70216

**Published:** 2026-10-23

**Authors:** Christopher Milde, Laura S. Brinskelle, Marcel Wilhelm, Julia A. Glombiewski

**Affiliations:** ^1^ Pain and Psychotherapy Research Lab, Department of Psychology RPTU Kaiserslautern‐Landau Landau Germany; ^2^ Department of Clinical Psychology and Psychotherapy Philipps‐Universität Marburg Marburg Germany

## Abstract

**Background:**

Unreliable pain reporting predicts placebo responses in clinical trials and has been documented in chronic pain populations. The thermal Focused Analgesia Selection Test (FAST) quantifies Pain Reporting Reliability (PRR) by assessing consistency when rating repeated thermal stimuli of varying intensities presented in pseudo‐randomized order. However, FAST's validity as a pure measure of reporting reliability remains unclear.

**Methods:**

We investigated whether PRR derived from the thermal FAST is confounded by individual differences in pain sensitivity (pain and tolerance thresholds), stimuli extending beyond individual pain ranges, and temporal trends. Fifty‐five healthy participants (32 women) completed the thermal FAST protocol comprising 49 stimuli across seven temperatures (43°C–50°C).

**Results:**

Higher pain thresholds predicted lower PRR (*b* = −4.66, 95% HDI [−9.18, −0.07]). Exploratory analysis suggests that rating variability was reduced when stimulation fell outside individual pain ranges (below‐range: *b* = −8.04, 95% HDI [−11.44, −4.72]; above‐range: *b* = −4.45, 95% HDI [−7.98, −0.90]). Greater coverage of FAST temperatures by individual pain ranges correlated with higher PRR (*ρ* = 0.33). Thermode repositioning to prevent habituation at the stimulation site may have induced acute sensitization at the repositioned site as pain ratings increased by +33 and +19 VAS points at the first two stimuli following repositioning. Excluding these trials improved median PRR by 16% (from *R* ^*2*^ = 0.65 to 0.81).

**Conclusions:**

PRR derived from the current thermal FAST implementation likely conflates reporting consistency with pain sensitivity, psychophysical range effects, and procedural artifacts and may therefore risk misclassifying pain phenotypes as reporting inconsistencies. We propose methodological refinements, particularly individualized stimulus calibration and optimized repositioning protocols, which should be evaluated in confirmatory studies and chronic pain populations.

## 1 Introduction

Pain assessment poses a fundamental challenge in clinical research and practice: it relies entirely on subjective report, yet treatment decisions, clinical trial outcomes, and our understanding of pain mechanisms depend critically on the reliability of these reports. The consequences of unreliable pain reporting are substantial: Inconsistent pain reporting contributes substantially to failure rates in clinical pain trials, where pharmacological interventions fail to demonstrate efficacy over placebo [1, 2, 3]. This variability also predicts greater placebo analgesia [2, 4] and associates with pain chronification, highlighting its relevance to both clinical trial methodology and chronic pain pathophysiology.

Recognizing this challenge, Treister et al. [5] developed the Focused Analgesia Selection Test (FAST) to systematically quantify Pain Reporting Reliability (PRR). The FAST estimates PRR by having individuals repeatedly rate pain intensity in response to noxious stimuli of varying physical intensity [6]—mechanical pressure [2] or thermal stimulation [5, 7]—presented in pseudo‐randomized order. This approach provides an experimental analogue to clinical pain diaries [4], with demonstrated correlations between PRR and clinical pain reporting consistency [5]. Moreover, FAST‐derived PRR negatively predicts placebo analgesia and training interventions that improve PRR have been shown to reduce placebo analgesia substantially [2], supporting a causal relationship between reporting reliability and placebo susceptibility.

Despite these promising applications, several methodological concerns suggest the thermal FAST may not provide a pure measure of reporting reliability. First, the thermal FAST uses a wide range of stimulation temperatures, including intensities frequently perceived as non‐painful or at tolerance thresholds. For thermal pain, individual pain ranges typically span from 45°C to 50°C in healthy populations [8, 9, 10]. Consequently, stimulation temperatures that fall below or exceed these individual ranges may produce floor or ceiling effects, artificially constraining rating variability and inflating apparent reliability estimates. Second, repeated thermal stimulation may induce habituation at lower intensities or sensitization at higher intensities across stimulation trials, introducing systematic temporal trends independent of reporting consistency. Third, practice effects may enhance rating consistency across stimulation trials, conflating learning with inherent reliability.

These confounds raise a critical question: Does the thermal FAST truly isolate PRR, or does it reflect a composite of rating ability, individual pain sensitivity, and methodological artifacts? Addressing this question is essential for valid interpretation of PRR in clinical trials, placebo prediction, and pain mechanism research. If FAST‐derived PRR confounds reporting consistency with pain sensitivity or paradigm‐related factors, its clinical and research applications require substantial reconsideration.

In this study, we examined potential confounds of the thermal FAST and derived recommendations toward more valid PRR measures. Specifically, we investigated whether the thermal FAST provides a valid measure of pain reporting reliability (PRR), or whether it conflates reliability with pain sensitivity and procedural artifacts. We hypothesized that individual pain and tolerance thresholds systematically affect FAST‐derived PRR (Hypotheses 1A and 1B) and that variability in pain ratings would be reduced at the lowest (43°C, 44°C) and highest (49°C, 50°C) stimulation temperatures (Hypotheses 2A and 2B). Additionally, we explored whether temporal trends related to habituation, sensitization, or practice effects systematically biased PRR estimates.

## 2 Methods

### 2.1 Participants

Healthy student volunteers were recruited through university mailing lists, social media, and oral advertisement. All participants provided written informed consent prior to participation. Psychology students received course credit, while other participants entered a voucher raffle. Personalized feedback on pain reporting performance was offered to all participants upon request.

Exclusion criteria assessed via self‐report included: local skin damage at the stimulus site (non‐dominant forearm), acute or chronic pain conditions, polyneuropathy, diabetes mellitus; acute psychiatric disorders, current analgesic use, pregnancy, substance use disorders, and obesity (BMI ≥ 30 kg/m^2^; World Health Organization, 2000).

Sample size was determined a priori using Monte Carlo power simulation (*α* = 0.05, power = 80%) based on an anticipated effect size of *r* = 0.40, yielding a target sample of *N* = 41. This effect size estimate was derived from weighted means reported in Pollatos et al. [11] who examined associations between interoceptive sensitivity and experimentally assessed pain thresholds and tolerance, and Weiss et al. [12], who investigated relationships between interoceptive accuracy, self‐regulation, and pain perception in healthy participants. To further explore differential sex effects and accommodate for potential exclusions due to data quality issues (e.g., constant pain ratings), we recruited 60 participants (deviating from the preregistered sample size).

Of 60 enrolled participants, five were excluded: three for BMI > 30 kg/m^2^, one for pregnancy discovered after enrolment, and one for undisclosed psychiatric condition. The final analyzed sample comprised 55 participants (32 women, 23 men; age: *M* = 23.98 years, SD = 4.38, range = 19–38; BMI: *M* = 22.67 kg/m^2^, SD = 3.12, range = 16.37–29.76), Table 1.

**TABLE 1:** Sample characteristics.

Variable	*N*	*M*/%	SD	Range
Age (years)	55	23.98	4.38	19–38
BMI (kg/m^2^)	55	22.67	3.12	16.37–29.76
Sex				
Female	32	58.2		
Male	23	41.8		

*Note:* For categorical variables, the *M* column reports the percentage.

Abbreviations: BMI, body‐mass‐index, M, mean; SD, standard deviation.

### 2.2 Procedure

Prior to laboratory assessment, participants completed an online survey SoSci‐Survey [13] containing demographic questions, psychological measures, and screening for exclusion criteria. During the laboratory session, participants were familiarized with thermal stimuli and the pain rating procedure using three thermal stimuli of varying intensity, see (Supplementary S1). The experimental protocol comprised: (1) baseline assessment of pain and tolerance thresholds, (2) the FAST protocol to quantify PRR, and (3) post‐FAST assessment of pain and tolerance thresholds. All thermal stimuli were delivered using a Peltier‐based contact thermode (30 × 30 mm; Thermal Sensory Analyzer TSA II, Medoc, Israel) applied to the volar surface of the non‐dominant forearm.

### 2.3 Pain Sensitivity Assessment

Pain and tolerance thresholds were determined using the method of limits [14]. Participants terminated an ascending thermal ramp (1°C/s, from 3°C baseline) via mouse click at two criterion points: when the stimulus first became painful (pain threshold) and when maximum tolerable pain was reached (tolerance threshold). Each threshold was assessed four times; the first trial served as familiarization, and the subsequent three trials were averaged to establish stable estimates. Threshold assessments were performed immediately before and after the FAST protocol to assess potential sensitization or habituation effects from repeated noxious stimulation. Measurement of tolerance thresholds was limited by the thermode's safety temperature of 50.5°C—implying that the actual tolerance threshold could not be measured in participants where the safety limit was reached.

### 2.4 Focused Analgesia Selection Test (FAST)

PRR was quantified using the thermal FAST paradigm [5]. The protocol comprised 49 thermal stimuli (7 temperatures × 7 repetitions): 43°C, 44°C, 46°C, 47°C, 48°C, 49°C, 50°C. Stimuli were organized into seven blocks, with each block containing one stimulus at each temperature. Temperature order was pseudo‐randomized within each block to minimize anticipatory effects.

Each stimulus followed a standardized temporal profile: ramping from 32°C baseline to the target temperature, maintained peak temperature for 3 s, then returning to baseline (total duration: 8 s). Ramp rates varied by target temperature (range: 4.4°C/s–7.2°C/s). Inter‐stimulus intervals ranged from 10 to 20 s to allow sufficient time for pain rating and thermal recovery.

To reduce local habituation or sensitization at a single skin site, the thermode was re‐positioned to an adjacent location on the forearm after every 14 stimuli (i.e., following completion of blocks 2, 4, and 6).

All stimuli were verbally announced prior to delivery to ensure predictability and reduce anticipatory anxiety, which can confound pain perception [15, 16]. Participants rated pain intensity after each stimulus using a computerized 100‐mm visual analogue scale (VAS) anchored at 0 (‘no pain’) and 100 (‘worst pain imaginable’).

### 2.5 Operationalization of Pain Reporting Reliability (PRR)

PRR was quantified by fitting Stevens' power law [17] to the stimulus–response relationship for each participant: $\text{Pain}=a*\text{Temperatur}e^{b}$. To ensure mathematical validity of the power function, temperatures were rescaled by subtracting the minimum FAST temperature (42°C) and adding 1, providing a strictly positive scale. We employed a two‐stage fitting procedure: First, log‐transforming both stimulus intensity and pain ratings linearizes the power law: $\log\left(\text{Pain}\right)=\log\left(a\right)+b*\log\left(\text{Temperature}\right)$. Ordinary least squares regression on the log–log transformed data provided initial parameter estimates for the Steven's power law with $a_{\text{start}}=e^{\text{intercept}}$ and $b_{\text{start}}=\text{slope}$. Second, these starting values initialized non‐linear least squares optimization (using the *nls* function in R) fitting the untransformed power law directly to the original pain ratings. We used the port algorithm with bounded constraints (*a* > 0.001, *b* > 0.001) to ensure physiologically plausible positive parameters and robust convergence.

PRR was operationalized as the coefficient of determination (*R* ^*2*^) from power function regression of pain ratings on stimulus temperatures, representing the proportion of rating variance explained by the stimulus–response function (0 = no relationship; 1 = perfect consistency). For all analyses where *R* ^*2*^ served as the outcome variable, values were multiplied by 100 to express percent variance explained, facilitating interpretation of regression coefficients.

### 2.6 Statistical Analysis

Data were analyzed using R (Version 4.5.1, R Core Team 2025) implemented in RStudio (Version 2025.9.1.401). Data visualization and pre‐processing were conducted using tidyverse [18].

#### 2.6.1 General Approach

Unless otherwise noted, analyses used pain and tolerance thresholds assessed before FAST administration to avoid confounding from FAST‐induced changes in pain sensitivity. All models were fitted using brms [19]. Continuous predictors were z‐standardized to facilitate prior specification and model convergence [20].

Models were estimated using Hamiltonian Monte Carlo with the No‐U‐Turn sampler (4 chains, 2000 warmup, 3000 post‐warmup iterations; 12,000 posterior samples). Default brms priors were used. Convergence was verified via *R̂* < 1.01 and effective sample sizes > 2000.

Evidence was evaluated via 95% highest density intervals (HDI) and probability of direction (pd) [21]. We interpreted pd > 95% as “substantial”, pd > 97.5% as “strong”, and pd > 99% as “very strong evidence” [22].

### 2.7 Preliminary Analysis: Temperature Discrimination

To verify participants discriminated FAST temperatures, we specified a Bayesian multilevel model with pain ratings predicted by temperatures with consecutive contrasts. A three‐level structure was specified: ratings within blocks within participants.

### 2.8 Primary Hypotheses

#### 2.8.1 Hypothesis 1 (Pain Sensitivity and PRR)

Separate models tested whether PRR (*R* ^*2*^ on a 0–100 scale) was predicted by (1A) pain threshold, or (1B) tolerance threshold.

#### 2.8.2 Hypothesis 2 (Pain Sensitivity and Rating Variability at Extreme Temperatures)

For each participant, we computed rating variability (SD across 7 repetitions) at 43°C and 44°C separately, then averaged these values to obtain within‐temperature variability at low FAST temperatures; the same procedure was applied at high temperatures (49°C, 50°C). We tested whether (2A) *pain thresholds* predicted low‐temperature rating variability and (2B) *tolerance thresholds* predicted high‐temperature rating variability.

### 2.9 Exploratory Analyses

#### 2.9.1 Floor/Ceiling Effects

For each participant, we computed rating variability at each FAST temperature. Bayesian multilevel models (with random intercepts for participants) contrasted rating variability (SD) at extreme temperatures (low: 43°C–44°C; high: 49°C–50°C) versus central temperatures (46°C–48°C; reference category).

#### 2.9.2 Individual Pain Range Effects

Each FAST temperature was classified relative to the participant's pain and tolerance threshold (below pain threshold, within pain range, above tolerance threshold). A Bayesian multilevel model (random intercepts for participants) contrasted rating variability at below‐range and above‐range temperatures versus within‐range temperatures (reference category).

#### 2.9.3 Temporal Trends

Graphical exploration revealed increased pain ratings at the initial stimuli (trials) of blocks following thermode repositioning (blocks 3, 5, 7, and initial block 1), independent of temperature.

To test this repositioning effect, we fitted a Bayesian linear mixed model with pain ratings predicted by temperature (ordered factor), block (ordered factor: 1–7), within‐block trial position (ordered factor: 1–7), and a repositioning factor (repositioning blocks: 1, 3, 5, 7; non‐repositioning: 2, 4, 6). Trial position and repositioning factor were modeled as an interaction to test whether temporal trajectories differed between block types. The model included random intercepts and slopes for trial position by participant, plus random intercepts for blocks within participants (three‐level hierarchical structure).

### 2.10 Intraclass Correlation Coefficient (ICC) as Complementary Reliability Measure

Post hoc, we complemented the *R* ^*2*^ measure of PRR by computing intraclass correlation coefficients (ICC) reflecting trial‐to‐trial consistency independent of power regression model fit. Person‐specific ICC values were derived from a Bayesian multilevel model in which pain intensity ratings were nested within stimulus temperatures within participants. ICC was defined as the proportion of total variance attributable to systematic between‐temperature differences relative to within‐temperature noise:

$$\mathrm{IC}C_{i}=\frac{\sigma_{\text{between}-\text{temperatures}}^{2}}{\sigma_{\text{between}-\text{temperatures}}^{2}+\sigma_{\text{within}-\text{temperatures}}^{2}}$$

Person‐specific estimates were extracted from the posterior distribution of random effects providing a posterior mean ICC per participant. To assess convergent validity, ICC and *R* ^2^ were Pearson correlated. To test whether ICC reliability was also affected by pain sensitivity, we conducted a beta‐regression predicting ICC from *z*‐standardized pain thresholds and tolerance thresholds in separate models.

### 2.11 Transparency and Openness

The study protocol was preregistered (https://osf.io/gr5zj) and ethical approval was obtained from the institutional review board (LEK‐369; 8th‐Dec‐2021).

## 3 Results

### 3.1 Pain Sensitivity

Unless otherwise noted, all analyses report pain and tolerance thresholds assessed before FAST administration. Mean pain threshold was 45.0°C (SD = 2.9, range = 38.1°C–50.5°C) and mean tolerance threshold was 49.1°C (SD = 1.8, range = 41.9°C–50.5°C). Both were strongly correlated (*r* = 0.65, 95% CI [0.46, 0.79]), indicating that individuals with higher pain thresholds also tended to tolerate higher temperatures.

Sex differences were found for tolerance: men (*M* = 50.0°C, SD = 1.0) tolerated higher temperatures than women (*M* = 48.4°C, SD = 2.0; *t*(47.9) = −3.79, *p* < 0.001, *d* = −0.98), representing a large effect. Pain thresholds did not significantly differ between men (*M* = 45.5°C, SD = 2.7) and women (*M* = 44.5°C, SD = 3.1; *t*(53) = 1.24, *p* = 0.221, *d* = −0.34), see (Supplementary S2).

#### 3.1.1 Ceiling Effects in Tolerance Assessment

Tolerance assessment was constrained by the thermode's 50.5°C safety limit, which 18 participants (32.7%; 14 men, 4 women) reached. These participants might have tolerated even higher temperatures if available, resulting in right‐censored tolerance estimates likely attenuating association strength. We therefore focus interpretation on pain threshold findings.

### 3.2 Temperature Discrimination in the FAST

Mean pain ratings monotonically increased across FAST temperatures, ranging from 6.77 VAS points (SD = 13.56) at 43°C to 68.15 VAS points (SD = 27.69) at 50°C, confirming the expected stimulus–response relationship.

Consecutive contrasts confirmed that participants successfully discriminated between adjacent FAST temperatures: 44 vs. 43°C (*Δ* = 2.89, 95% HDI [0.21, 5.64]), 46 vs. 44°C (*Δ* = 9.54, 95% HDI [6.84, 12.29]), 47 vs. 46°C (*Δ* = 6.55, 95% HDI [3.67, 9.13]), 48 vs. 47°C (*Δ* = 9.27, 95% HDI [6.47, 11.85]), 49 vs. 48°C (*Δ* = 14.40, 95% HDI [11.63, 17.11]), and 50 vs. 49°C (*Δ* = 18.77, 95% HDI [16.07, 21.46]). The smallest effect occurred at the lowest temperatures (43 vs. 44°C), potentially reflecting floor effects where many participants reported minimal or no pain at both temperatures.

### 3.3 Primary Hypotheses

#### 3.3.1 Effect of Pain Sensitivity on Pain Reporting Reliability

Mean PRR was *R* ^*2*^ = 0.61 (Median = 0.65, SD = 0.17), representing 61% variance explained—closely replicating Treister et al.'s [5] original validation study (*M* = 0.61, SD = 0.16).

### 3.4 Hypothesis 1A—Pain Thresholds and PRR

Supporting our hypothesis, higher pain thresholds related to lower PRR (*b* = −4.66, 95% HDI [−9.18, −0.07], pd = 97.8%; Figure 1A1,A2), indicating that participants perceiving the FAST temperatures as less painful showed less consistent pain reporting. Each SD increase in pain thresholds (2.9°C) was related to a 4.66% decrease in PRR.

**FIGURE 1:**
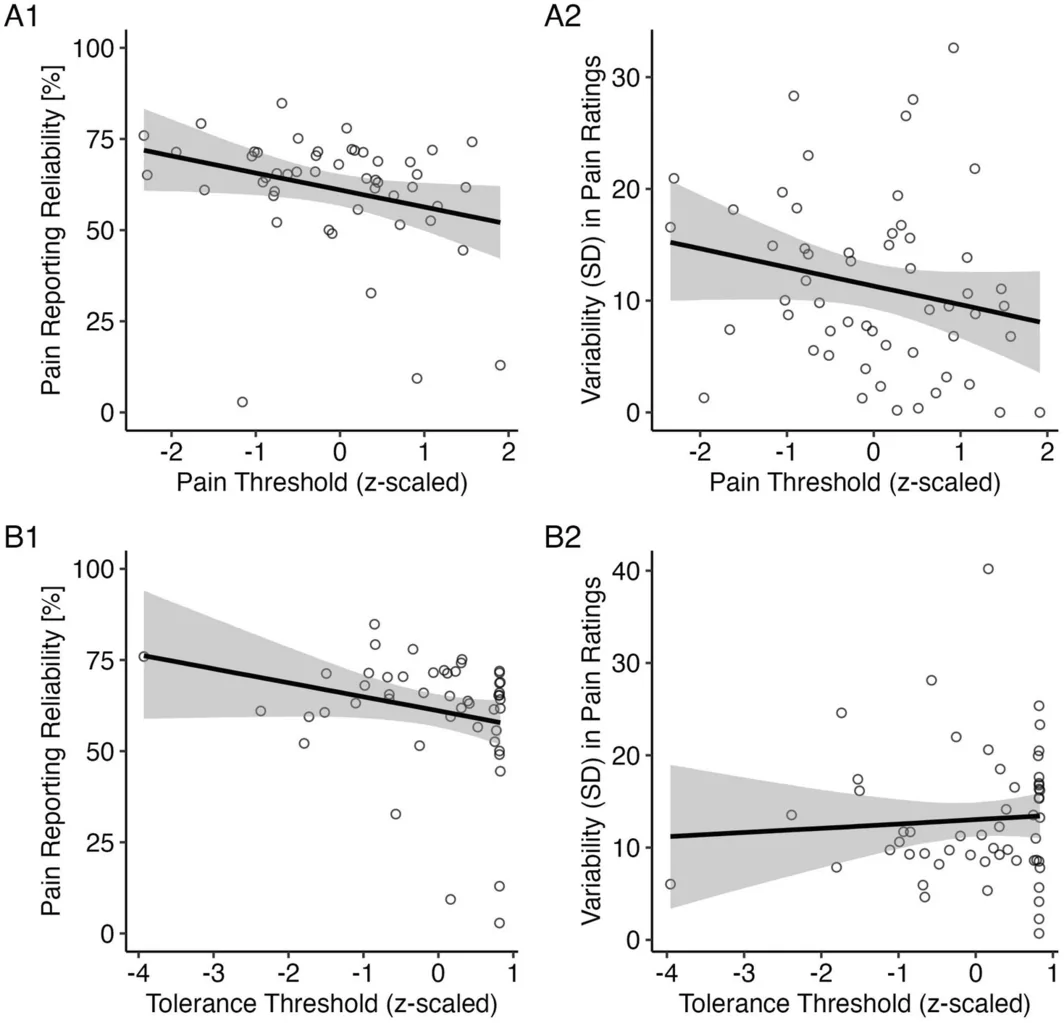
Prediction of Pain Reporting Reliability (PRR) and variability (SD) in pain ratings by pain thresholds (A1, A2), tolerance thresholds (B1, B2). Shown are slopes with 95% highest density intervals. Open circles indicate individual observations. PRR corresponds to *R* ^*2*^ expressed as % explained variance in power regression fits.

### 3.5 Hypothesis 1B—Tolerance Thresholds and PRR

Similarly, higher tolerance predicted lower PRR (*b* = −3.84, 95% HDI [−8.32, 0.63], pd = 95.6%; Figure 1B1,B2). However, the 95% HDI included zero, indicating substantial uncertainty in this estimate. Ceiling effects (32.7% of the participants reached the thermode's safety limit) likely obscured this relationship, artificially truncating the distribution and attenuating observable relationships.

### 3.6 Effect of Pain Sensitivity on Rating Variability at Extreme Temperatures

#### 3.6.1 Hypothesis 2A—Pain Thresholds and Low Temperature Rating Variability

We found inconclusive evidence that higher pain thresholds predicted reduced rating variability at the lowest FAST temperatures (43°C–44°C; *b* = −1.67, 95% HDI [−3.81, 0.42], pd = 94.3%; Figure 2A). The HDI included zero, and pd fell below our 95% threshold for substantial evidence, precluding conclusive evidence.

**FIGURE 2:**
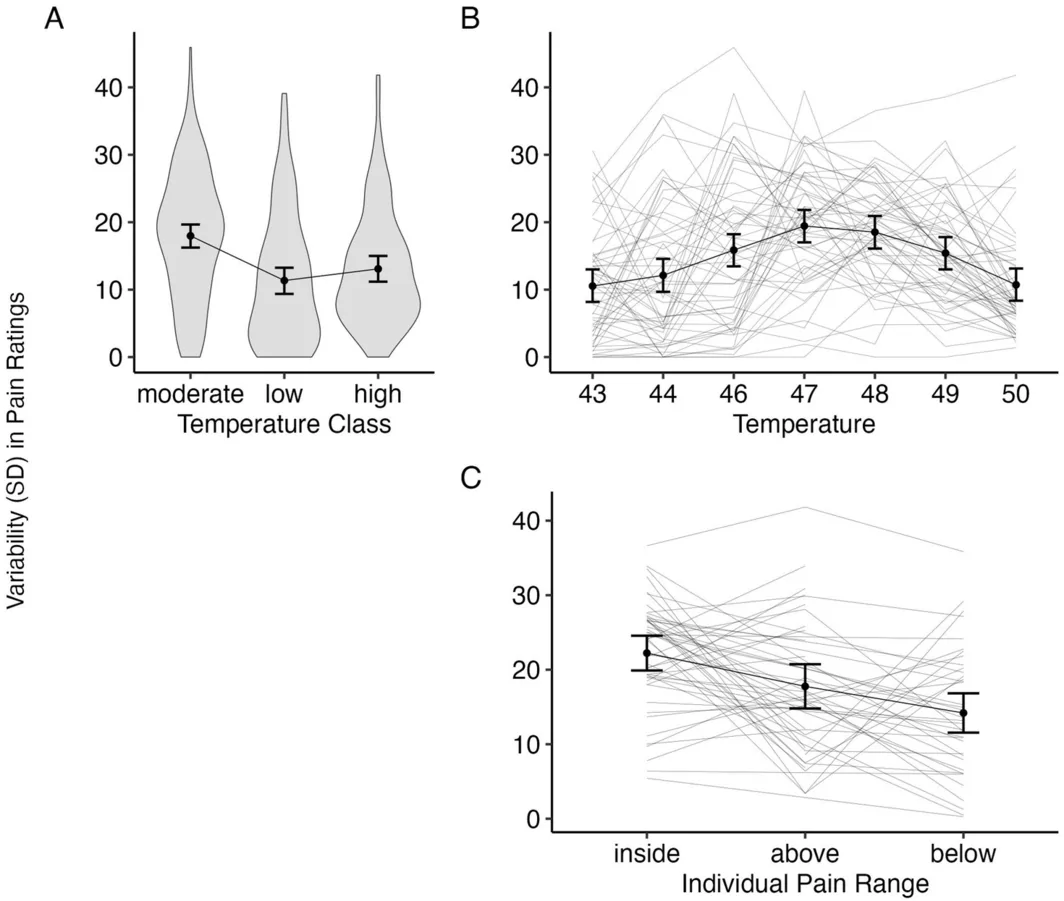
Variability in pain intensity ratings across stimulation temperatures of the FAST: (A) Variability (SD) in pain ratings at the two lowest (43°C and 44°C) and the two highest (49°C and 50°C) temperatures compared to the central temperatures. (B) Rating variability across each temperature. (C) Rating variability at temperatures that where inside, above or below the individuals' pain range. Shown are posterior means with 95% highest density intervals.

#### 3.6.2 Hypothesis 2B—Tolerance Thresholds and High Temperature Rating Variability

We found no evidence that higher tolerance predicted reduced rating variability at the highest FAST temperatures (49°C–50°C; *b* = 0.46, 95% HDI [−1.34, 2.45], pd = 68.9%; Figure 2B). As with Hypothesis 1B, tolerance ceiling effects due to the thermode's safety limit might have obscured any existing relationship.

### 3.7 Exploratory Analyses

#### 3.7.1 Floor and Ceiling Effects at Extreme Temperatures

Visual inspection of individual stimulus–response curves showed systematically reduced rating variability at the extreme ends of the FAST temperature range, see (Supplementary S3). To test whether this pattern held at the group level, we compared rating variability at extreme temperatures versus central temperatures. We found very strong evidence for rating variability reduction at both the lowest FAST temperatures (43°C–44°C; *b* = −6.63 VAS points, 95% HDI [−8.57, −4.71], pd > 99%) and the highest temperatures (49°C–50°C; *b* = −4.90 VAS points, 95% HDI [−6.80, −2.96], pd > 99%) compared to central temperatures (46°C, 47°C, 48°C; Figure 2A).

Figure 2B illustrates this U‐shaped pattern of rating variability across all seven FAST temperatures, with maximum variability at central temperatures (*M* = 18.0 VAS points, SD = 9.48) and marked reductions at both the two lowest (*M* = 11.4 VAS points, SD = 9.53) and two highest stimulation temperatures (*M* = 13.1 VAS points, SD = 8.32).

#### 3.7.2 Individual Pain Range Effects on Rating Variability

On average, 33.1% of stimuli fell below pain thresholds, 46.7% within pain range, and 20.3% above individual tolerance thresholds. We found very strong evidence for reduced rating variability when stimuli fell outside participants' pain ranges: Rating variability was reduced both below pain thresholds (*M* = 14.0 VAS points, SD = 8.7; *b* = −8.04, 95% HDI [−11.44, −4.72], pd > 99%) and above tolerance (*M* = 18.0 VAS points, SD = 10.0; *b* = −4.45, 95% HDI [−7.98, −0.90], pd > 99%) compared to within‐range temperatures (*M* = 22.2 VAS points, SD = 7.1; Figure 2C). These effects represent substantial reductions in rating variability—approximately 37% reduction at temperatures below pain threshold and 19% reduction at temperatures above the individual's pain range. Consistent with these range‐dependent effect on rating variability, greater coverage of FAST temperatures by individual pain ranges was related to higher *R* ^*2*^ values (*ρ* = 0.33, *p* = 0.026, 95% CI [0.03, 0.58]).

### 3.8 Repositioning Artifacts

#### 3.8.1 Repositioning Effects

Pain ratings spiked following thermode repositioning to an adjacent skin site (blocks 1, 3, 4, 7), independent of FAST temperature (Figure 3A). At trial position 1, ratings increased by 33.1 VAS points (95% HDI [29.6, 36.7], pd > 99%) in repositioning versus non‐repositioning blocks. At trial position 2, the increase was +18.6 points (95% HDI [15.0, 22.2], pd > 99%) and at trial position 3, the increase was +3.7 points (95% HDI [0.17, 7.4], pd = 97.5%; Figure 3B).

**FIGURE 3:**
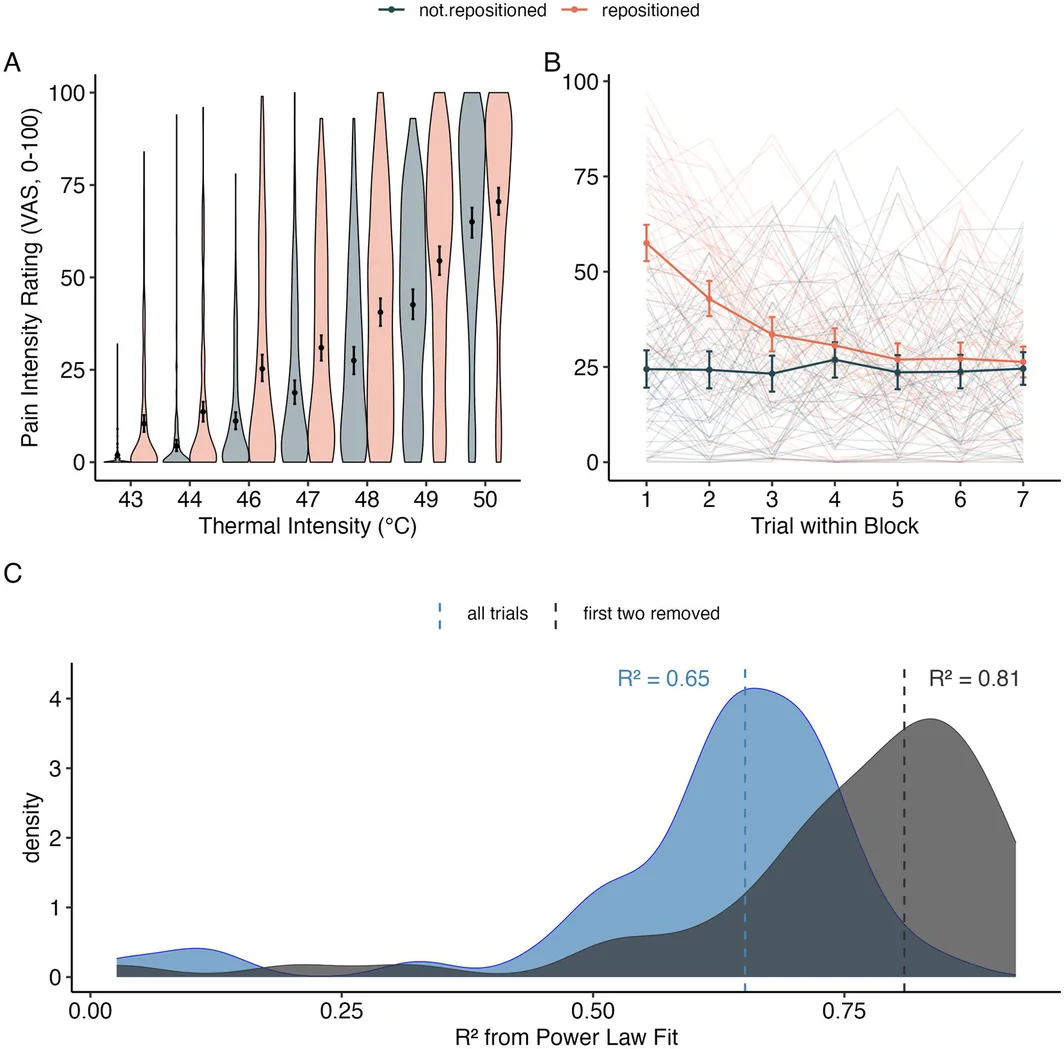
Repositioning effect of the thermode. (A) Sensitization at blocks when the thermode was moved to an adjacent skin site (blocks: 1, 3, 5, 7). Shown are violin plots overlaid with means with 95% confidence intervals. (B) Sensitization at the initial within‐block trials after repositioning the thermode to an adjacent skin site. Shown are posterior means with 95% highest density intervals. Thin lines indicate participant trajectories. (C) Improved Pain Reporting Reliability (PRR) when accounting for the repositioning effect (power regressions were computer after excluding first two trials in repositioning blocks). Shown are median *R* ^*2*^ values of PRR to account for the skewed distributions.

### 3.9 Impact of Repositioning on PRR Estimation

This repositioning‐induced sensitization artificially inflates rating variability by introducing systematic variance unrelated to participants' intrinsic reporting ability. Excluding the first two trials after each repositioning (8 trials in total), median PRR increased from *R* ^*2*^ = 0.65 to *R* ^*2*^ = 0.81—a 16% improvement (Figure 3C). Mean PRR increased from *R* ^*2*^ = 0.61 (SD = 0.17) to *R* ^*2*^ = 0.75 (SD = 0.18), and the PRR‐distribution shifted substantially right‐ward, with fewer participants showing poor reliability (*R* ^*2*^ < 0.40).

### 3.10 Intraclass Correlation Coefficient (ICC) as Complementary Reliability Measure

The median ICC reflecting trial‐to‐trial consistency was 0.62 (*M* = 0.58, SD = 0.15, range = 0.16–0.75). Both reliability measures (*R* ^*2*^ and ICC) showed high convergent validity for both the non‐adjusted *R* ^*2*^ (*r* = 0.77, 95% CI [0.62, 0.86]). Consistent with the associations found between *R* ^*2*^ and pain sensitivity (see Primary Hypotheses), both pain thresholds (*b* = −0.17, 95% HDI [−0.33, −0.01], pd = 98.3%) and tolerance thresholds (*b* = −0.22, 95% HDI [−0.40, −0.06], pd = 99.5%) were related to lower ICC values.

## 4 Discussion

This study evaluated whether the thermal Focused Analgesia Selection Test (FAST) provides a valid measure of Pain Reporting Reliability (PRR). Our findings suggest that FAST‐derived PRR does not purely reflect an individual's ability to report pain consistently but may be confounded by three factors: individual differences in pain sensitivity, psychophysical floor and ceiling effects at extreme FAST temperatures, and methodological artifacts from thermode repositioning. These confounds might challenge the validity of the current thermal FAST for its proposed applications in clinical trial design, placebo prediction, and chronic pain assessment. Critically, the confounds identified here are properties of the thermal implementation of the FAST—we make no claims about the validity of the FAST when using other stimulation modalities.

### 4.1 Systematic Confounds in PRR Measurement

#### 4.1.1 Pain Sensitivity Confounds

Supporting Hypothesis 1A, individuals with higher pain thresholds showed reduced PRR (4.7% decrease in PRR per SD increase in threshold). This contradicts the intuitive assumption that reduced variability indicates higher rating consistency. Instead, when FAST temperatures fall below individual pain thresholds (as 43°C–44°C frequently did for higher‐threshold participants), the ratings concentrate on or near zero. This likely restricts rating variability, which in turn reduces PRR estimates independently of actual reporting ability.

This interpretation aligns with psychophysical theory: the power regression model underlying *R* ^*2*^ estimation assumes graded responses across the full stimulus range [23]. However, when several stimuli fall outside an individual's pain range, this assumption is violated. This reduces the model's fit and artificially depresses PRR. Critically, this reduction occurs independently of the actual reporting consistency and could therefore reflect a systematic confound rather than a genuine difference in reporting ability. Current thermal FAST protocols do not account for these individual differences in pain sensitivity when deriving PRR estimates [2, 5].

Importantly, we found evidence that pain sensitivity affected different measures of pain reporting reliability as similar effects were found for trial‐to‐trial consistency assessed with ICC—suggesting that the effects of interindividual differences in pain sensitivity is not specific to *R* ^2^‐based PRR but may affect PRR measures in thermal FAST protocols more broadly.

#### 4.1.2 Psychophysical Range Effects

Our exploratory analyses suggest that rating variability was reduced both at extreme FAST temperatures and—more importantly—at any temperature falling outside individual pain ranges (36% reduction below pain threshold and 20% above tolerance threshold). This pattern is consistent with the interpretation that the confound originates from the mismatch between fixed stimulus ranges and individual pain sensitivity rather than from specific stimulation temperatures.

It could be assumed that risk of bias in PRR by pain sensitivity manifests differently in chronic pain populations: In the present non‐clinical sample, the lowest FAST temperatures were often perceived as non‐painful, and the highest FAST temperature was often below the actual tolerance threshold of the participant. In chronic pain populations, however, these floor effects might be less pronounced, as temperatures of 43°C or 44°C are more likely to be perceived as painful. Regarding ceiling effects, it could be assumed that thermodes' safety limit is less often reached; however, tolerance thresholds in some populations at affected stimulation sites might be well below the maximum temperature of the thermal FAST.

#### 4.1.3 Methodological Artifacts: Repositioning Effect

Our exploratory analyses further indicate a “repositioning effect”: pain ratings were elevated by 33 VAS points at the first trial following thermode relocation, independent of stimulus temperature. Notably, excluding the first two post‐repositioning trials increased median PRR by 16% (from 65% to 81%), indicating that a substantial portion of measured “unreliability” reflects procedural artifacts rather than inconsistent reporting.

This finding aligns with evidence that thermode repositioning produces simultaneous site‐specific habituation (preventing progressive response decrements at a single location) and site‐nonspecific sensitization (transiently augmenting responses at adjacent skin sites) [24]. While repositioning successfully prevents habituation, it simultaneously introduces acute sensitization. Participants might have accurately detected genuine pain intensity differences caused by peripheral sensitization at the repositioned skin site. Consequently, variability attributed to poor rating reliability may actually reflect valid detection of protocol‐induced sensitization effects. The magnitude of this effect might indicate that thermal FAST‐derived PRR estimates are highly contingent on specific protocol design choices such as repositioning procedures, which could limit comparability across studies.

### 4.2 Toward Valid PRR Measurement

These findings suggest various methodological refinements that should be evaluated:

1. Individualized stimulus calibration. Rather than fixed temperatures, calibrate temperatures to each participant's pain and tolerance thresholds, ensuring all stimuli fall within their pain range. This approach, standard in psychophysical paradigms [14, 25] could reduce floor and ceiling effects by matching stimulus ranges to individual pain sensitivity.
2. Optimized repositioning protocol. Maintain repositioning to prevent habituation, but introduce additional stimuli immediately after relocation and exclude the first 2–3 trials from PRR estimation. Alternatively, increase inter‐stimulus intervals following repositioning to allow complete sensitization dissipation.
3. Alternate assessment approaches. Consider mechanical pressure stimuli [2]; mechanical pressure stimuli have demonstrated good‐to‐excellent test–retest reliability [26] and may provide relatively stable measurements across repeated trials, whereas thermal measures—particularly stimulus–response functions—can show lower reliability [27]. Alternatively, topical capsaicin could increase heat pain sensitivity [28] avoiding absence of pain at low FAST‐temperatures and ceiling effects for tolerance thresholds due to the thermode's 50.5°C safety limit.

Importantly, the effects of these refinements should be assessed also in targeted clinical populations that are expected to differ in pain phenotypes such as pain sensitivity or adaptation to thermode repositioning.

### 4.3 Clinical and Research Implications

Clinical trials frequently fail to demonstrate treatment efficacy over placebo due to high response variability and associated preferential placebo responding [29, 30]. PRR has been proposed as a screening tool to identify—and potentially exclude—preferential placebo responders [2]. Because FAST‐derived PRR was associated with pain sensitivity in our sample, trial selection based on the current thermal FAST might risk excluding participants based on their pain sensitivity rather than their reporting reliability. This could paradoxically reduce trial generalizability or inadvertently enrich samples with specific pain profiles, potentially obscuring true treatment effects.

The observed negative association between PRR and placebo analgesia [2] may partly reflect pain sensitivity confounds rather than pure reporting effects. If individuals with higher thresholds receive lower PRR scores due to floor effects rather than genuine unreliability, observed PRR‐placebo relationships could be mediated by threshold‐related factors. An optimized thermal FAST that isolates true reporting reliability from pain sensitivity confounds could provide clearer mechanistic insights into placebo responding and potentially enhance predictive validity.

Methodologically refined PRR measurement could advance chronic pain research, where pain‐nociception decoupling [31, 32] and fluctuating daily pain reports are common [29, 30]. However, investigating whether low PRR constitutes a genuine risk factor for pain chronification [4, 33], requires measures that reflect reporting reliability rather than interindividual differences in pain sensitivity. Similarly, assessing whether FAST‐training improves pain outcomes via reporting consistency [2]—similar to sensory discrimination trainings [32, 34]—or by alternative mechanisms demands a construct‐valid PRR measure as the basis for inference.

Refined PRR operationalization could also provide clearer tests of predictive processing models of pain and placebo analgesia [35, 36, 37], which propose that precise nociceptive signal detection reduces expectation‐driven analgesia [4]. Such theoretical advances presuppose, however, that PRR indexes the intended construct of reporting reliability rather than sensitivity‐related variance—an assumption that requires careful empirical validation before these frameworks can be meaningfully tested.

Should future work establish construct validity of an optimized PRR measure, FAST‐derived PRR might eventually contribute to personalized treatment approaches [4]. Low PRR has been associated with chronic pain characteristics, including reduced pain‐nociception coupling [31], deficits in sensory discrimination, and increased variability in daily pain reports [29, 38, 39]. Whether FAST‐based training enhances pain outcomes [40, 41] or attenuates fear generalization [32, 42] via improving pain reporting consistency remains a promising but speculative direction that awaits validation in clinical samples assessed with construct‐valid PRR protocols.

### 4.4 Limitations

Several methodological factors warrant consideration. The thermode's 50.5°C safety limit created ceiling effects in tolerance assessment, particularly for men (32.7% reached the safety limit), constraining characterization of pain ranges in high threshold individuals. However, this limitation strengthens our core finding; even with this constraint, we detected substantial reduction in rating variability at the highest FAST temperatures that compromise PRR estimation. A thermode without safety limits would likely reveal even more pronounced confounding.

We employed the method of limits for threshold assessment, which may introduce reaction‐time biases [25, 43]. However, such biases would be consistent across participants and unlikely to explain the systematic threshold‐PRR relationships observed. Minor protocol deviations from Treister et al. [5]—using 50°C rather than 51°C maximum and 44°C rather than 45°C as second‐lowest temperature—were implemented to prevent tissue damage while maintaining equal temperature spacing for the two lowest and two highest stimulation temperatures, consistent with recent FAST applications [6].

The analyses of psychophysical range and repositioning effects were exploratory and conducted in a non‐clinical sample of moderate size; therefore, they require confirmation in independent, preregistered studies.

## 5 Conclusion

The FAST addresses a critical need in pain research: quantifying PRR to improve assay sensitivity in clinical trials and understand the role of pain variability in predicting placebo responses and pain chronification. However, our findings suggest that the current thermal FAST implementation may conflate true reporting consistency with pain sensitivity, psychophysical range mismatches, and procedural artifacts. We propose methodological refinements—individualized stimulus calibration and optimized repositioning protocols—that could yield valid PRR estimates. These refinements should be tested to assure that the thermal FAST reliably distinguishes individuals based on genuine reporting inconsistencies rather than differences in pain sensitivity. Valid PRR measurement would be an important prerequisite for using the FAST to improve assay sensitivity in clinical trials and could advance our mechanistic understanding of pain variability in chronic pain and placebo responsiveness. Future work should test optimized thermal FAST protocols, incorporating individualized calibration and control of procedural artifacts, in clinical pain populations to assess their validity and utility for predicting clinically relevant outcomes. Because sexes differ substantially in pain sensitivity [44, 45], differences in pain sensitivity and how they affect FAST‐derived measures of pain reporting reliability should be further investigated in larger samples.

## Author Contributions

C.M.: Study Conceptualization, Data Curation, Data Analysis, Writing of the Original Draft, Supervision; L.S.B.: Data Acquisition, Data Analysis, Writing of the Original Draft; M.W.: Manuscript Revision; J.A.G.: Study Conceptualization, Manuscript Revision.

## Funding

This research was supported by the Research Initiative of Rhineland‐Palatinate, Germany (Forschungsinitiative Rheinland‐Pfalz).

## Ethics Statement

Ethical approval was obtained from the institutional review board (LEK‐369; 8th‐Dec‐2021).

## Consent

Written informed consent was obtained from all participants.

## Conflicts of Interest

The authors declare no conflicts of interest.

## Supporting information


**Supplementary S1:** Study procedure flow chart.
**Supplementary S2:** Sex‐specific differences in pain and tolerance thresholds. Shown are violin plots with mean and 95% confidence intervals grouped by threshold (pain and tolerance) and sex (female and male). Individual trajectories are shown as lines.
**Supplementary S3:** Exemplary participants showing reduced rating variability outside the individuals' pain range. Rating variability showed floor effects when stimulation temperatures were smaller than the individuals' pain threshold and ceiling effects when temperatures were greater than the individuals' tolerance threshold. Pain ranges are indicated as gray shaded area. The dashed line indicates the power fit curve.

## Data Availability

The data that support the findings of this study are available from the corresponding author upon reasonable request.
