# Supplementary material for: Limitations in the Assessment of Pain Reporting Reliability—Validation of the Focused Analgesia Selection Test
Source: Pain Pract. 2026 Oct 23;26(8):e70216. doi: 10.1111/papr.70216 (PMC13692801; doi:10.1111/papr.70216)
Supplement: Supplementary file 1 [file PAPR-26-0-s001.docx]

**Supplementary Material**

**Online-Survey**

- Socio-demographical data
- Depression: PHQ-15 (Gräfe et al., 2004)
- Depression: ADS-K (Meyer & Hautzinger, 2001)
- Somatization: PHQ-7 (Gräfe et al., 2004)
- Interoceptive Awareness: MAIA-2 (Mehling et al., 2018)
- Neuroticism: NEO-FFI (Costa & McCrae, 1992)
- Pain Anxiety: PASS-20 (Kreddig et al., 2015)
- Trait Anxiety and Depression: STADI-T (Laux et al., 2016)
- Exclusion criteria
- Study information and informed consent

**Familiarization with the FAST**

- 3 heat pain stimuli (45°C,43°C,47°C)
- 3 pain intensity ratings

**Determination of Pain Sensitivity (t1)**

Method of limits

- 4 ascending stimuli for pain threshold
- 4 ascending stimuli for tolerance threshold

**Determination of Pain Reporting Reliability (PRR)**

FAST

- 49 heat pain stimuli
- 7x7 pain intensity ratings

**Determination of Pain Sensitivity (t2)**

Method of limits

- 4 ascending stimuli for pain threshold
- 4 ascending stimuli for tolerance threshold

**Supplementary 1** Study procedure flow chart.

**Supplementary 2** Sex-specific differences in pain and tolerance thresholds. Shown are violin plots with mean and 95% confidence intervals grouped by threshold (pain and tolerance) and sex (female and male). Individual trajectories are shown as lines.

**Supplementary 3** Exemplary participants showing reduced rating variability outside the individuals’ pain range. Rating variability showed floor effects when stimulation temperatures were smaller than the individuals’ pain threshold and ceiling effects when temperatures were greater than the individuals’ tolerance threshold. *Note:* Pain ranges are indicated as grey shaded area. The dashed line indicates the power fit curve.
